# Catalyzing a Reproductive Health and Social Justice Movement

**DOI:** 10.1007/s10995-015-1917-5

**Published:** 2016-01-06

**Authors:** Sarah Verbiest, Christina Kiko Malin, Mario Drummonds, Milton Kotelchuck

**Affiliations:** School of Social Work and the Center for Maternal and Infant Health, University of North Carolina at Chapel Hill, Campus Box 7181, Room 3018 Old Clinic Building, Chapel Hill, NC 27599-7181 USA; National Preconception Health and Health Care Initiative, Chapel Hill, NC USA; Alameda County Public Health Department, 1000 Broadway, Suite 500, Oakland, CA 94607 USA; Northern Manhattan Perinatal Partnership, Inc., 127 West 127th Street, 3rd Floor, New York, NY 10027 USA; Division of General Academic Pediatrics, Maternal and Child Health MGH, Center for Child & Adolescent Health Research and Policy, Mass General Hospital for Children, Pediatrics Harvard Medical School, Boston, MA USA

**Keywords:** Preconception, Life course, Reproductive equity, MCH leadership, Health equity, Infant mortality, Social determinants of health

## Abstract

*Objectives* The maternal and child health (MCH) community, partnering with women and their families, has the potential to play a critical role in advancing a new multi-sector social movement focused on creating a women’s reproductive and economic justice agenda. Since the turn of the twenty-first century, the MCH field has been planting seeds for change. The time has come for this work to bear fruit as many states are facing stagnant or slow progress in reducing infant mortality, increasing maternal death rates, and growing health inequities. *Methods* This paper synthesizes three current, interrelated approaches to addressing MCH challenges—life course theory, preconception health, and social justice/reproductive equity. *Conclusion* Based on these core constructs, the authors offer four directions for advancing efforts to improve MCH outcomes. The first is to ensure access to quality health care for all. The second is to facilitate change through critical conversations about challenging issues such as poverty, racism, sexism, and immigration; the relevance of evidence-based practice in disenfranchised communities; and how we might be perpetuating inequities in our institutions. The third is to develop collaborative spaces in which leaders across diverse sectors can see their roles in creating equitable neighborhood conditions that ensure optimal reproductive choices and outcomes for women and their families. Last, the authors suggest that leaders engage the MCH workforce and its consumers in dialogue and action about local and national policies that address the social determinants of health and how these policies influence reproductive and early childhood outcomes.

## Significance

This article synthesizes core constructs, historical challenges, and current opportunities for maternal and child health leaders in addressing the complex and tenacious challenge of maternal and infant mortality and morbidity. The paper offers four strategic directions that have the potential to foster innovation and change, and serve as a roadmap to leaders within and beyond MCH. The authors call for a new social movement to create an agenda for women’s reproductive health and economic justice that will usher in a new period of health, social, and financial development for women, girls, and their family members.

## Introduction

Since the turn of the twenty-first century, the field of maternal and child health (MCH) has been planting seeds for significant change. These efforts have been guided by a new paradigm offered by the life course theory, which has widened our lens to look more broadly at infant and maternal mortality and morbidity as a social barometer with an intergenerational impact. This approach requires moving beyond a singular focus on clinical interventions to addressing issues that affect the health and well-being of young women and men, including poverty, racism, education, violence, and reproductive justice. Preconception health has been synchronized with this approach since the release of national recommendations and goals in 2006, which identified the importance of reproductive awareness, life planning, and comprehensive services for young women and men [7]. Because the United States is facing stagnant or slow progress in reducing infant mortality, increasing maternal death rates, and growing health disparities, we offer directions for our MCH colleagues to consider as we work collectively and creatively to improve the health and wellness of our nation’s mothers, fathers, and families. This paper aims to spark innovation and new connections within and outside of the MCH field and to engage critical partners in this endeavor across public health, social work, social justice, reproductive justice, business, and community development.

## Core Constructs for Change

Work by Lu and Halfon [8] built on the biopsychosocial framework and applied the expanded framework across a life course model [8]. This work opened up new ways of not only linking health and wellness across the life span, but also tracking the relentless impact of social inequity as it layers on itself over time to create shorter life expectancies and limited life opportunities for disproportionately affected populations. The growing body of research in epigenetics demonstrates that a person’s life trajectory begins with the health of his or her parents and their social conditions. Further, the life course model opens discussion for creating services and programs that not only serve young children but also serve families raising young children. Moreover, this model creates connections to fields such as chronic disease by providing a framework to explain the link between high-risk pregnancies, poor birth outcomes, and less-than optimal health later in life. As the MCH field continues to struggle with 2.5 fold racial disparities in birth outcomes, the life course model elucidates how the social determinants of health, described as protective and risk factors, led to these inequities. Lu et al.’s [9] work on the *12 Point Plan for Closing the Black White Gap in Birth Outcomes* offers a roadmap for action in the domains of improving health care, strengthening families and communities, and addressing social and economic inequities.

Although evidence of the impact of a woman’s health prior to pregnancy on birth outcomes has been available for decades, the promotion of preconception health only began gaining momentum in the MCH field since 2006. This momentum was prompted by the release of recommendations to improve preconception health and health care in the United States by the Centers for Disease Control and Prevention/ATSDR Work Group and the Select Panel on Preconception Health. Narrowly defined, preconception care is a set of interventions that endeavor to identify and modify biomedical, behavioral, and social risks to a woman’s health or pregnancy outcome through prevention and management. However, the national movement has consistently broadened this definition to encompass reproductive life planning, access to comprehensive and quality health care, high reproductive awareness for both women and men, and the elimination of disparities in health outcomes [7]. A greater understanding of the importance of women’s wellness and reproductive health in reducing infant mortality has pushed the MCH field to expand its focus beyond immediate pregnancy and early childhood interventions to new areas that include women’s and men’s preventive health services, reproductive life planning, and maternal care for up to 2 years postpartum. The HRSA Maternal and Child Health Bureau has begun to align major programs such as Healthy Start and Title V to address this larger framework. Likewise, the Office of Population Affairs partnered with the Centers for Disease Control and Prevention (CDC) to introduce *ProvidingQuality Family Planning Services* for the Title X program and public and private providers of comprehensive primary care, guidelines that include preconception health services for women and men [5].

Such efforts have been informed by a growing urgency to address both the conditions in which people live—and children are raised—and differential access to quality health care, education, and career opportunities, especially for people of color. Health equity is achieved when all people have the opportunity to attain their full health potential regardless of their race/ethnicity, income, education, or other social circumstances [3]. Work by Hogan et al. [6] issued a call to action for public health to spotlight health equity. The authors challenged leaders to do more than simply learn about the life course model and catalogue disparities, but to act to change internal policies and strategies that will shift the ways in which services are delivered so that young women and men of color are less likely to encounter barriers to the services they need to be well and determine their life plans. Notably, the authors emphasized that decades of institutionalized racism require intentional efforts not only to eliminate barriers but also to augment services to close gaps in health and life expectancy [6].

Combined, these seeds of change require public health leaders to take a holistic view of women’s wellness and reproductive health that recognizes the role social, economic, and political factors play in influencing the conditions and environments in which women and men make decisions about their reproductive health and futures. Optimal conditions for making these decisions should be experienced equally by all women and men in our country. Preconception health does not imply that we must provide a narrowly defined, isolationist set of services to women based on the assumption that they will have children someday; rather, this perspective urges that special consideration should be given to the importance of the preconception period because this life stage can have a profound impact on the health of women, men, and any children they choose to have.

## Challenges to this Work

Moving these frameworks and constructs into action is not a small task. The MCH field has developed expertise, programs, and funding streams built around prenatal care and core MCH services and their related performance metrics. Although the MCH field recognizes the importance of connecting with colleagues in other disciplines, including chronic disease, family planning, health equity, infectious disease, housing, transportation, economic development, and environmental health, these connections are developing slowly, largely because these groups also function within specific funding streams and areas of expertise. Moreover, even though these fields serve the same populations and have similar goals as MCH, few models exist of fully integrative practices with resource sharing across these partners.

Preconception health has offered an opportunity to broaden the MCH perspective, emphasizing that the field needs a generation of healthy young adults to have significant improvements in birth outcomes and children’s health and well-being. Historically, there has been power in leveraging the role of maternal health in moving policy forward for women’s wellness. However, the belief that preconception health is focused only on a woman’s reproductive function (maternalism) is narrow and incorrect [14]. At its core, preconception health supports family planning, thereby assuring that women and men can become parents if and when they want to, and that they have the opportunity to be as healthy as possible before having a child. Ongoing national and state political debates and actions that question funding for the full suite of family planning options (e.g., long-acting reversible contraceptives, emergency contraception, and abortion) create a challenging backdrop against which public health leaders, clinicians, and women must function.

Further, preconception health must include men. Men’s health and their role in supporting the health and well-being of their children and partners are important. Engaging men in preconception health requires new ways of thinking about how we conduct intakes, provide services, and manage complex issues around relationships. Giving more attention to thinking about men’s sexual health and reproductive planning might move some people beyond their comfort zone, but this wider view is essential to achieving reproductive justice.

## Opportunities

Historical cycles have shown a convergence of key factors make conditions ripe for social change. We believe the current period of US social history is in such a cycle, with key factors in play that create fertile ground for a new movement for reproductive health and social justice. First, the Affordable Care Act has brought significant disruption to the health care system. As access to care has increased, the health care industry has experienced increasing pressure to provide cost-efficient, equitable population-based care to newly insured populations and to address health disparities. With this shift, hospitals and clinics are being held accountable for health outcomes influenced by factors outside their doors, requiring that these medical centers build partnerships and conduct community outreach in new ways. Further, health care reform has introduced a business rationale for prevention because steps toward universal coverage create higher stakes for payers in ensuring the health of populations, particularly young men and women. Efforts have been underway since 2012 to enroll people in health insurance, to collect stories from the newly insured and people who have been denied services, and to push states to expand coverage for all residents. Health care reform is reshaping the identity of public health and its role in ensuring the health of communities. Policy makers and health care leaders are increasingly aware of the impact of social determinants on health and life opportunities, which in turn offers public health the chance to leverage its influence to elevate innovative strategies for change.

Activists are engaged on many issues. Movements such as Black Lives Matter provide an opening for discussion on complex issues and create an important opportunity for MCH professionals to help frame and shape these conversations. Vocal calls for a living wage for all workers and equal pay for equal work have also seen resurgence. Significant strides are being made in advancing the civil rights of the lesbian, gay, bisexual, transgender, and questioning (LGBTQ) community, particularly in regard to marriage equality. Moreover, America’s ongoing inability to address racism, power, and privilege have been front and center on newspaper pages, particularly regarding the over-policing of young men of color, incarceration, and violence directed at places of worship. Now is an opportune time to turn up the volume on the dialogue about the impact of economics, environment, and opportunity on the health and stability of communities by specifically considering how these factors affect the well-being of women, children, fathers, and families.

## The Way Forward

Given these constructs, challenges, and opportunities, what is the role of MCH professionals in making sure all people have the social, political, and economic power necessary to make decisions about their health, reproduction, and future? The MCH community, partnering with women and their families, has the potential to play a critical role in advancing a new multi-sector social movement focused on creating a women’s and men’s reproductive and economic justice agenda. Given that the majority of people in the communities served by public health and social justice entities have children, will have children, have had children, support or live with children, or are children (including adolescents and youth), extensive opportunities exist to infuse an MCH perspective into the work done by non-MCH partners. A key goal of preconception health and the life course approach is to build agency for all women and men to make decisions that will ensure good reproductive health. This goal can best be accomplished by creating neighborhood conditions that support good reproductive decisions, resulting in healthy children who will become healthy adults and populate healthy communities. To begin this work, we offer four strategic directions for consideration and conversation.

### Strategic Direction 1: Stay the Course for Access to Quality Health Care for All

Young women and men cannot achieve their optimal health without access to coordinated, comprehensive care, including preventive screenings, health education, medical services, contraceptives, behavioral health care services, and dental care. The capacity to afford these services through universal health insurance is a big step, but one that many states have not yet made. As seen in the 2014 National Women’s Health report [1], wide variations exist across states in health coverage for women, women’s access to health care, and women’s health outcomes. Universal access to health care is imperative.

Young adults also need care that facilitates access (e.g., evening and weekend services) and services provided in a culturally-competent manner. Further, all people, regardless of their language, race, ethnicity, or geography, should receive evidence-based care and have the information needed to be full partners in their health care. For example, a recent study by the Center for Reproductive Rights [4] and SisterSong (a women of color reproductive justice collaborative) suggested that African American women living in Georgia and Mississippi—two states with among the highest maternal death rates in the country—experience key inequities, including poor quality sexual and reproductive health information and services, lack of access to needed reproductive care, and discrimination throughout the health care system.

MCH professionals can continue to support health care reform through multiple avenues, such as building innovative partnerships; implementing outreach efforts to enroll all eligible persons in insurance plans; supporting the provision of evidence-based, quality preventive services; and encouraging health care systems to understand and lean into their role in reducing health inequities. Moreover, MCH professionals have the opportunity to bolster the health care system with wrap-around community services, to encourage providers to ask their patients and clients about reproductive intentions, and to provide trauma-informed care paired with access to comprehensive behavioral health services as needed. Such opportunity also entails a unique responsibility for critical developmental periods outlined by the life course approach, including late adolescence as teens enter legal adulthood, new mothers in their postpartum year, and young adults who might not think they need preventive health services.

### Strategic Direction 2: Facilitate Change through Crucial Conversations and Listening Sessions

Although meetings will not change the world, intentional dialogue that allows for listening and sharing can change hearts, minds, and perspectives. Few opportunities exist for diverse groups of colleagues or partners to deeply and honestly discuss critical and challenging issues such as racism, power structures, privilege, sexism, immigration, and reproductive equity. Such dialogue is crucial and deserves time and attention. The queries suggested by Hogan et al. [6] including “What is the public health community doing to actively disassemble processes that feed inequity in our own institutions?” (p. 1148) are a good place to begin. Supporting quality conversations requires leaders who are aware of their own perspectives and biases and are well-versed in facilitating courageous conversations. Encouraging such dialogue also requires a broad agency commitment to responding to recommendations that emerge from these conversations and calls for changes in practice and internal systems.

As we engage in courageous conversations, all voices should be heard—not just the voices of formal leaders, but also the voices of front-line workers, young professionals, and consumers whose perspectives are essential to providing authentic, effective, and innovative services. MCH professionals need to take lead roles in engaging people outside public health in the dialogue to ensure diverse perspectives are included from social workers, community developers, health care administrators, women’s studies leaders, business leaders, social entrepreneurs, reproductive justice leaders, and faith leaders. When done well, dialogues across differences (including political or religious lines) can be powerful and can lead to new program and policy directions.

As MCH leaders, we understand that specific protocols exist to establish “evidence” and then share evidence in public health and other fields. This approach is largely a construct that continues to support an academic/clinical professional power structure made up of individuals conducting biomedical research and publishing results in journals that most communities cannot readily access. We cannot ignore one of the most obvious and effective ways to make change: asking women and their communities what they need and how they want to engage with MCH professionals to knock down barriers and create programs with an increased chance of sustainability. We have to rely on women and men to tell us their stories, needs, hopes, and ideas for program and policy changes in order to transform the reproductive and women’s health landscape in America.

The MCH community has the skills needed to facilitate this conversation—via social media, qualitative research, well-constructed staff and consumer satisfaction surveys, and taking the time to talk with women waiting for services. Listening and agenda-building sessions should take place in every city and state; the policy recommendations and action-oriented tasks that arise should be documented, prioritized, assigned, advocated for, and included in budgets. Conversations among women’s groups that represent diverse backgrounds should be supported to generate common ground on a few issues and then use that consensus to better mobilize women as a critical voting block on key issues. This strategy can create political will and innovative solutions. These conversations are necessary if we are to spark change, build community, and approach our work in ways that are more efficient and effective.

### Strategic Direction 3: Develop Collaborative, Comprehensive Programs within and Beyond Public Health that Support Preconception Health, Reproductive Equity, and Life Planning

Set within a life course context, preconception health and reproductive life planning not only recognize the importance of healthy infants, but also acknowledge the equitable physical, social, educational, and economic development of the woman, her partner, and her larger family. The MCH footprint transcends traditional silos. Therefore, our charge is to create a sense of shared value in and commitment to the belief that, at its most basic level, reproductive health equity is the foundation of a healthy community.

Numerous programs and initiatives are underway across the country that support reproductive health equity by addressing the social determinants of health. These programs have great potential. For example, the Promise Zones program designates high-poverty urban, rural, and tribal communities as Promise Zones where the federal government will partner with and invest in communities to create jobs, leverage private investments, increase economic activity, expand educational opportunities, and improve public safety [13]. The Best Babies Zone (BBZ), funded by the W.K. Kellogg Foundation, is a place-based approach to reducing infant mortality. BBZ uses the concept of small neighborhood zones to engage residents and local community organizations in identifying opportunities for collaborative action that will improve neighborhood conditions so everyone can thrive. The BBZ serves as a catalyst and convener to bring together resources with community vision to create neighborhood-led initiatives that link health services, early care and education, economic development, and community systems [2]. The BBZ model does not focus on doing work *to* a community but rather on working in partnership *with* the community on projects that are resident-designed and led. The Robert Wood Johnson Foundation’s focus on *Building a Culture of Health* [11] also embraces a multifaceted “whole community” approach to health.

A collective impact effort to improve reproductive health could be achieved by marshalling the expertise, resources, and networks of other public health sectors, including chronic disease, infectious diseases, tobacco prevention and control, community clinics, health equity policy, planning, environmental health, violence prevention, and early childhood education. “It is imperative to combine and deploy the scientific, social, and programmatic development resources of the public health community to lead the charge in creating a reproductive health equity roadmap for all stakeholders” [6, p. 1148]. Life course theory creates a space for public health leaders to work across sectors to better engage diverse partners in this effort, while maintaining a sharp focus on the importance of preconception health and reproductive planning as a shared endeavor. Intermediate steps can be taken to expand the MCH imperative into the purview of these sectors, beginning with cross-informing and strategically using existing resources to model incremental, multi-sector programmatic change.

### Strategic Direction 4: Educate and Engage the MCH Workforce and Its Consumers on Local and National Issues that Address the Social Determinants of Health: Demonstrate the Impact of These Policies on Improved Reproductive Health and Birth/Early Childhood Outcomes

Key social determinants of health positively affect the life course of women, men, and young families, and include factors such as equitable pay, living wages, workplace policies, access to affordable health insurance, access to affordable, safe housing, local food movements, educational pipelines of young children (birth through age 8 years), college affordability and completion, civic engagement, just policing, and access to affordable quality childcare. An opportunity for new leadership has come forth from the emergence of social media and its power and the convergence of the positive disruption brought by health care reform, street protests, and growing voices calling for greater attention on social determinants of health and equity. As Petersen [10] commented.

Leadership is about creating and communicating a shared vision for a changing future and while one of the bulwark features of MCH is its endurance… it is the vision that endures while the means and the methods of achieving that vision evolve over time, populations and circumstances (p. 245).

Significant movements focused on social determinants of health would benefit from engaging with MCH professionals and consumers. The MCH field can engage in this important work by describing the clear connections these efforts have with preconception health, life course, and reproductive health equity. To make a meaningful contribution, the MCH workforce must commit to learning about these issues, especially in terms of state and local efforts and policy. In addition, MCH professionals would benefit from training in adaptive leadership, which mobilizes people to tackle difficult root causes of a problem. Challenging and innovative work is required if we are to capitalize on our current moment in time. To achieve this goal, it will be critically important that graduate students and professionals across the career continuum receive training in communication, creative conflict, social entrepreneurship, and how to construct “unusual” partnerships.

For example, we can begin immediately by focusing on protecting the political voting power of women, students, and people of color. As part of our MCH work, we must ensure, in a nonpartisan manner, that all of our clients are registered to vote and are encouraged to exercise that right in local, state, and national elections. Clients should be reminded that every vote counts and that they should engage with and vote for candidates who represent their political, economic, and social interests. Our responsibility is to help clients understand that civic engagement is one way they can improve health, gain economic power, and achieve reproductive justice in America. We can find ways to promote voter registration and share nonpartisan information about the issues. We can ask candidates to share their opinions on key reproductive health and MCH issues during bipartisan debates and discussion forums.

What else can we do? We can foster community dialogue by screening thought-provoking documentaries such as *The Raising of America* (www.raisingofamerica.org) or *Unnatural Causes* (www.unnaturalcauses.org). MCH professionals can document the effects of lack of access to health care, lack of affordable childcare, and lack of sick leave on health outcomes. We can identify topics receiving attention in our community based on current events and policy challenges, and make a commitment to developing a depth of knowledge about at least one new topic area. In our personal time, we can engage other sectors in our lives (e.g., faith communities, clubs, sororities, leagues) in discussions on these issues. Using our skills in building partnerships, we can align with the efforts of other groups such as Moms Rising (www.momsrising.org) that are already working to bring awareness of these issues to women in all communities. Such joint efforts might include creating learning communities at work that meet over lunch to share information and perspectives on various issues. Further, as MCH professionals build new partnerships, we will collectively increase our leverage locally, statewide, and nationally to win transformational policy, program, and system changes.

Although it remains imperative that public health professionals respect the boundaries of their employers regarding speaking out at work, we might be restraining our voices more than necessary. Without crossing these boundaries, we can still raise topics in meetings; educate public officials about our issues; and demonstrate to county commissioners the positive or negative impact of local business practices, zoning laws, and public budgets on the health of women and children. We can use the *Health in All Policies* approach that forces leaders to evaluate if new policies and program initiatives move toward health equity or sustain inequality, whether inadvertently or intentionally [12]. We can support our clients and community members in building their capacity to contribute to critical conversations at city hall, health department board meetings, state legislatures, and beyond. In the same way that we hope other agencies and sectors will ally with us in supporting preconception health, reproductive life planning, and maternal and infant health, MCH professionals as a group must be willing to expand our own boundaries and knowledge to learn about economic and political issues that affect the daily lives and well-being of the people we care about.

## Conclusion

Maternal and child health is built upon the work of a group of women who applied their passion, skills, and evidence to create a national focus on children and families [10]. The time has come for a revival in our field. We believe that the MCH workforce should form alliances with other professionals in community development, social work, social justice, the faith community, and other arenas to create a new movement for our time: A movement that recognizes the impact of policy and privilege on the health of generations and engages a broad array of thought leaders and community advocates in creating change; an effort that can help actualize the vision of the National Preconception Health and Health Care Initiative that all women and men of reproductive age will achieve optimal health and wellness, fostering a healthy life course for them and any children they may have.

We challenge our colleagues and professionals in other fields to advocate relentlessly for high quality, comprehensive, culturally-appropriate health care for everyone. We believe a foundational aspect of public health advocacy is to train for and orchestrate conversations among colleagues and with communities about the core issues of our day, including racism, structures of power, gender discrimination, and privilege. Collaboration is not optional, nor is moving beyond our comfort zone and traditional organizations. Initiating a new social movement will require unconventional alliances and the capacity to engage more broadly to enhance the conditions where people eat, live, work, play, pray, and create families. As a profession, we have much to learn, and we hope that colleagues will take advantage of existing training opportunities and create new training opportunities where gaps exist. Further, we must fulfill our critical public health function of demonstrating the impact (via data and surveillance) of social changes on health trajectories.

Given that the vast majority of people in this nation have children, have had children, support or care for children, or are children, conversations about creating the conditions for healthy reproduction have implications for the majority of the population and future generations. These conversations widen the lens through which we view our work, creating space for a collective approach and a new language that unites the MCH field and moves us towards our vision for healthy women, men, children, families, and communities. The time is ripe for the next social movement to focus on creating a reproductive health and economic justice agenda that will usher in a new period of health, social, and financial development for women, families, and communities across America.
